# The Usefulness of Intravascular Ultrasound and Optical Coherence Tomography in Patients Treated with Rotational Atherectomy: An Analysis Based on a Large National Registry

**DOI:** 10.3390/jcdd11060177

**Published:** 2024-06-10

**Authors:** Wojciech Siłka, Michał Kuzemczak, Krzysztof Piotr Malinowski, Łukasz Kołtowski, Kinga Glądys, Mariola Kłak, Ewa Kowacka, Damian Grzegorek, Piotr Waciński, Michał Chyrchel, Miłosz Dziarmaga, Sylwia Iwańczyk, Miłosz Jaguszewski, Wojciech Wańha, Wojciech Wojakowski, Fabrizio D’Ascenzo, Zbigniew Siudak, Rafał Januszek

**Affiliations:** 1 Faculty of Medicine, Jagiellonian University Medical College, 31-008 Cracow, Poland; silkawojciech@gmail.com (W.S.); kinga.gladys64@gmail.com (K.G.); mchyrchel@gmail.com (M.C.); 2 Division of Emergency Medicine, Poznan University of Medical Sciences, 61-701 Poznan, Poland; michal.kuzemczak@gmail.com; 3 Department of Cardiology, Biegański Hospital, Medical University of Lodz, 91-347 Łódź, Poland; 4 Department of Interventional Cardiology and Internal Diseases, Military Institute of Medicine—National Research Institute, 05-119 Legionowo, Poland; 5 Faculty of Medicine, Department of Bioinformatics and Telemedicine, Jagiellonian University Medical College, 31-008 Cracow, Poland; krzysztof.piotr.malinowski@gmail.com; 6 Center for Digital Medicine and Robotics, Jagiellonian University Medical College, 31-008 Cracow, Poland; 7 1st Department of Cardiology, Medical University of Warsaw, 02-091 Warsaw, Poland; lukasz@koltowski.com; 8 Faculty of Medicine and Health Sciences, Andrzej Frycz Modrzewski Cracow University, 30-705 Cracow, Poland; mariola.klak@onet.pl (M.K.); ewakowacka90@gmail.com (E.K.); 9 Department of Cardiology, John Paul II Provincial Hospital, 97-400 Bełchatów, Poland; dajmon850@op.pl; 10 Department of Cardiology, Medical University of Lublin, 20-059 Lublin, Poland; 11 Department of Cardiology and Cardiovascular Interventions, University Hospital, 30-688 Cracow, Poland; 12 Department of Cardiology-Intensive Therapy and Internal Diseases, Poznan University of Medical Sciences, 61-701 Poznan, Poland; dziarmaga.milosz@spsk2.pl; 13 1st Department of Cardiology, Poznan University of Medical Sciences, 61-701 Poznan, Poland; syl.iwanczyk@gmail.com; 14 1st Department of Cardiology, Medical University of Gdansk, 80-210 Gdansk, Poland; mjaguszewski@gumed.edu.pl; 15 Department of Cardiology and Structural Heart Diseases, Medical University of Silesia, 40-055 Katowice, Poland; wojciech.wanha@gmail.com (W.W.); wwojakowski@sum.edu.pl (W.W.); 16 Division of Cardiology, University of Turin, 10-124 Turin, Italy; fabrizio.dascenzo@gmail.com; 17 Faculty of Medicine and Health Sciences, Jan Kochanowski University, 25-369 Kielce, Poland; zbigniew.siudak@gmail.com

**Keywords:** intravascular imaging, IVUS, OCT, rotational atherectomy, PCI

## Abstract

Background: Intravascular ultrasound (IVUS) and optical coherence tomography (OCT) have been shown to improve the clinical outcomes of percutaneous coronary interventions (PCIs) in selected subsets of patients. Aim: The aim was to investigate whether the use of OCT or IVUS during a PCI with rotational atherectomy (RA-PCI) will increase the odds for successful revascularization, defined as thrombolysis in myocardial infarction (TIMI) 3 flow. Methods: Data were obtained from the national registry of PCIs (ORPKI) maintained by the Association of Cardiovascular Interventions (AISN) of the Polish Cardiac Society. The dataset includes PCIs spanning from January 2014 to December 2021. Results: A total of 6522 RA-PCIs were analyzed, out of which 708 (10.9%) were guided by IVUS and 86 (1.3%) by OCT. The postprocedural TIMI 3 flow was achieved significantly more often in RA-PCIs guided by intravascular imaging (98.7% vs. 96.6%, *p* < 0.0001). Multivariable analysis revealed that using IVUS and OCT was independently associated with an increased chance of achieving postprocedural TIMI 3 flow by 67% (odds ratio (OR), 1.67; 95% confidence interval (CI): 1.40–1.99; *p* < 0.0001) and 66% (OR, 1.66; 95% CI: 1.09–2.54; *p* = 0.02), respectively. Other factors associated with successful revascularization were as follows: previous PCI (OR, 1.72; *p* < 0.0001) and coronary artery bypass grafting (OR, 1.09; *p* = 0.002), hypertension (OR, 1.14; *p* < 0.0001), fractional flow reserve assessment during angiogram (OR, 1.47; *p* < 0.0001), bifurcation PCI (OR, 3.06; *p* < 0.0001), and stent implantation (OR, 19.6, *p* < 0.0001). Conclusions: PCIs with rotational atherectomy guided by intravascular imaging modalities (IVUS or OCT) are associated with a higher procedural success rate compared to angio-guided procedures.

## 1. Introduction

In contemporary clinical practice, the procedural success rate of percutaneous coronary interventions (PCIs) exceeds 95% [1]. However, the overall short- and long-term clinical outcomes depend on multiple factors, including but not limited to the patient’s condition at baseline, medical history, and operator experience [2,3,4,5]. Furthermore, dynamic advances in interventional technologies along with older and more comorbid patients translate into more complex and challenging cases to deal with in a cath lab, which were previously not eligible for PCI and were usually referred for coronary artery bypass graft surgery [6,7,8].

In order to achieve a sufficient lumen gain translating into short- and long-term postprocedural coronary patency, proper lesion preparation as well as optimal stent positioning and sizing play a crucial role. Therefore, intravascular imaging modalities, namely intravascular ultrasound (IVUS) and optical coherent tomography (OCT), have been introduced into the clinical arena. Importantly, despite clear benefits of intravascular imaging, particularly in more complex lesions, the use of the above-mentioned techniques varies, but it is still insufficient in Europe and North America [9]. According to the reported data from the National Inpatient Sample, intravascular imaging is used in less than 10% of all PCI procedures performed in the United States [10]. European data vary from country to country. Despite a significant increase in the use of these techniques, the British data indicate that intravascular imaging is used in 18% of cases, while in Poland in 2021, it was only 4.42% [11,12]. The rate of OCT use rarely exceeds 1 percent in all-comers patients [10,13].

The usefulness of IVUS and OCT among patients undergoing PCI of calcified coronary lesions has been demonstrated in several studies [14,15,16,17]. RA is an effective technique for calcified plaques, facilitating stent delivery and implantation. While IVUS- and OCT-guided PCIs have been demonstrated to yield improved clinical outcomes in complex lesions compared to angiography-guided procedures, the association between the use of IVUS/OCT in RAs and angiographic success (defined as postprocedural TIMI 3 flow) in real-world patients has not been extensively elucidated. Importantly, some available data indicate that intravascular imaging findings may predict coronary flow disturbances following RA [18]. Therefore, in the present study, we aimed to evaluate whether the use of intravascular imaging modalities is independently associated with the angiographic success rate of PCI with concomitant RA.

## 2. Methods

### 2.1. Materials and Study Design

The present study is a retrospective analysis of prospectively collected data obtained from the Polish National Registry of Percutaneous Coronary Interventions (ORPKI). The registry has already been described in previously published papers [19,20]. Data were collected from the registry for PCIs performed between January 2014 and December 2021. We extracted 6522 PCIs performed with concomitant rotational atherectomy. Out of them, 788 (12.08%) PCIs were guided by intravascular imaging, either IVUS (708, 89.8%) or OCT (86, 10.91%). The technical aspects of the procedure, such as the access site, catheter size, type of a coronary guidewire, intravascular imaging modality and burr diameter, were left at the operator’s discretion. Patients were referred for RA-PCI and intravascular imaging according to the current European Guidelines [21,22,23,24]. The protocol complied with the Declaration of Helsinki, and all the patients gave written informed consent prior the coronary procedure. Due to the retrospective nature and anonymization of the collected data, obtaining the consent of the Bioethics Committee was not required.

### 2.2. Statistical Analysis

Continuous variables are presented as means [standard deviation] and medians [first quartile; third quartile]. Normality was assessed via the Shapiro–Wilk test or Kolmogorov–Smirnov test with Lilliefors correction for variables with more than 2000 observations. The differences between the 2 groups were compared using the Student’s or Welch’s *t*-tests, depending on the equality of variance for normally distributed variables. In case of nonparametric data, the Mann–Whitney U test was introduced. Categorical variables were compared with Pearson’s chi-squared or Fisher’s exact test if 20% of the cells had an expected count of less than 5 (Monte Carlo simulation for Fisher’s test using tables of dimensions higher than 2 × 2). All demographic and clinical patients’ characteristics were adopted as potential factors associated with successful revascularization in univariable logistic regression models. Variables with a *p*-value < 0.2 were included in the final multivariable model constructed using minimization of the Akaike Information Criterion in order to find factors linked to the postprocedural TIMI 3 flow achievement. Statistical analysis was performed using R version 4.1.1 (R Foundation for Statistical Computing, Vienna, Austria, 2021) with the ‘rms’ package, version 6.2-0.

## 3. Results

A total of 6522 patients undergoing RA-PCI were analyzed in this study. Out of them, 788 (12.1%) underwent PCI with the guidance of intravascular imaging, either OCT (10.9%) or IVUS (89.8%). The postprocedural TIMI 3 flow was achieved significantly more often in RA-PCIs performed with intravascular imaging compared to angiography-guided procedures (98.7% vs. 96.6%, *p* < 0.0001).

### 3.1. General Characteristics at Baseline

The baseline characteristics are presented in Table 1 and Table 2. Patients who underwent RA-PCI guided by intravascular imaging modalities were younger (*p* = 0.03), less often males (*p* = 0.01), and less frequently suffered from diabetes mellitus (*p* = 0.005). However, arterial hypertension was significantly more prevalent among patients undergoing OCT compared to those who underwent RA-PCI without OCT (*p* = 0.001). Patients who were treated through RA-PCI with the guidance of intravascular imaging presented with acute coronary syndromes more often, including unstable angina, STEMI, or NSTEMI. Both comparison groups were similar according to the Killip classification at presentation.

### 3.2. Vascular Access, Coronary Angiography, and Procedural Indices

The femoral approach was significantly more common in patients undergoing IVUS- or OCT-guided procedures, whereas radial access was more frequently used for RA-PCIs without intravascular guidance (*p* = 0.01, Table 3). With regard to vascular access, no difference was found between groups in the analysis restricted to OCT-guided procedures. Isolated LMCA disease and MVD were found more frequently in patients undergoing imaging-guided RA-PCIs, whereas SVD was more prevalent in angiography-guided procedures. Furthermore, bivalirudin was more often used during PCIs guided using OCT or IVUS (Table 3), whereas P2Y_12_ inhibitors were used more frequently during PCI performed without IVUS, i.e., either with OCT or without any intravascular imaging guidance (Table 3).

Bifurcation PCI was more common in intravascular imaging-guided procedures compared to angiography-guided PCIs (*p* < 0.001, Table 4). Patients treated with an intravascular imaging-guided RA-PCI had a lower rate of TIMI 0/1 prior to the procedure (14.3% vs. 19.6%, *p* < 0.001) as compared to subjects undergoing RA-PCIs without intravascular imaging guidance. Contrast dye volumes and radiation exposure were higher in IVUS- or ICT-guided procedures (Table 4).

### 3.3. Periprocedural Complications

There were no significant differences between the study groups for periprocedural complications rates including death, myocardial infarction, no-flow/no-reflow/slow-flow phenomena, access site bleeding, cardiac arrest, allergic reactions, and coronary artery perforation (Table 5).

### 3.4. Predictors of a Technical Success

**Figure 1:**
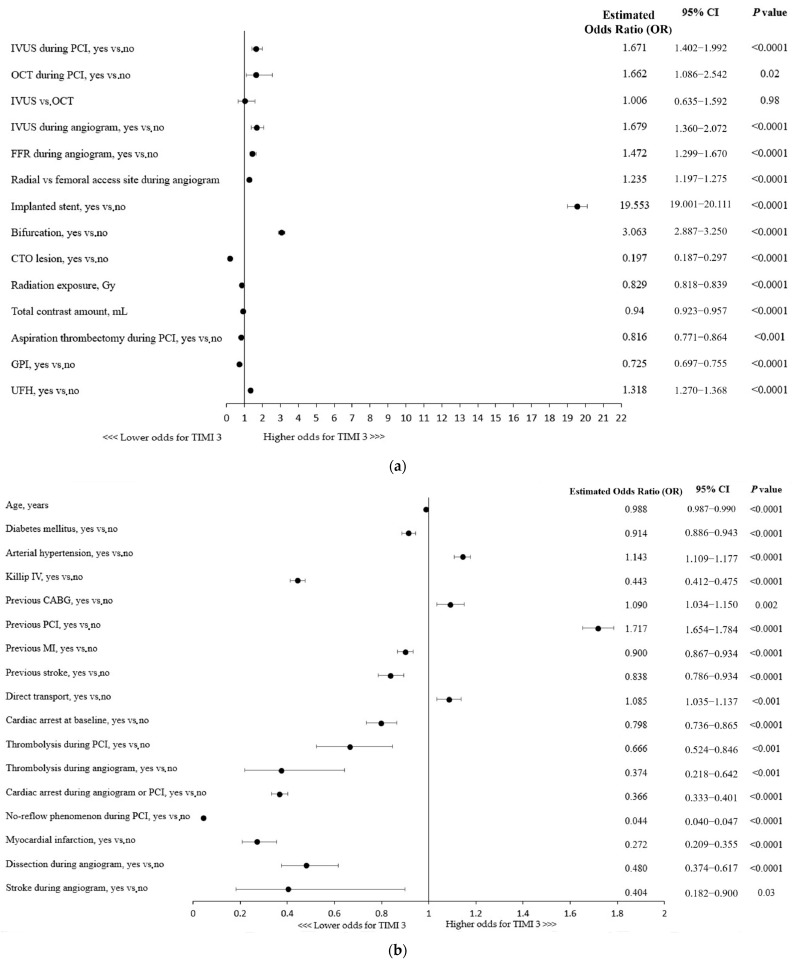

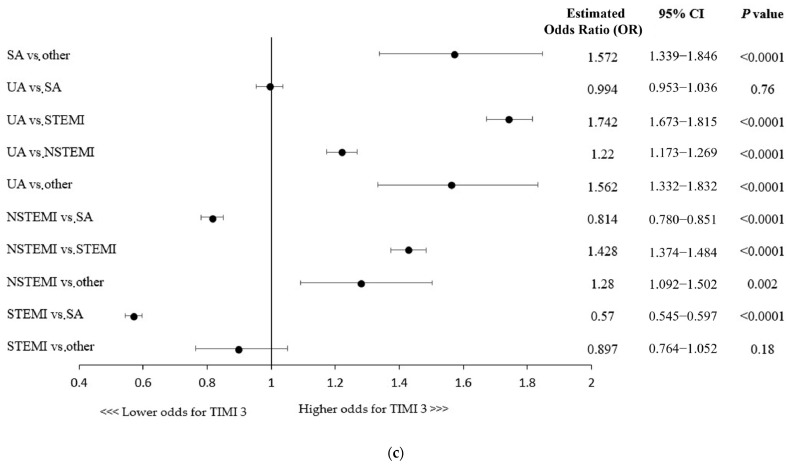
(**a**) Factors associated with thrombolysis in myocardial infarction (TIMI) 3 flow: multivariable analysis. CTO, chronic total occlusion; FFR, fractional flow reserve; GPI, glycoprotein IIb/IIIa inhibitors; IVUS, intravascular ultrasound; OCT, optical coherence tomography; PCI, percutaneous coronary intervention. (**b**) CABG, coronary artery bypass grafting; MI, myocardial infarction; PCI, percutaneous coronary intervention. (**c**) Factors associated with thrombolysis in myocardial infarction (TIMI) 3 flow with regard to patient clinical presentation: multivariable analysis. NSTEMI, non-ST-segment elevation myocardial infarction; SA, stable angina; STEMI, ST-segment elevation myocardial infarction; UA, unstable angina.

The use of any of the intravascular imaging modalities, IVUS (OR, 1.67; 95% CI: 1.40–1.99; *p* < 0.0001) or OCT (OR, 1.66; 95% CI: 1.09–2.54; *p* = 0.02), for the guidance of RA-PCI was independently associated with the technical success of the procedure, defined as TIMI 3 flow following the PCI (Figure 1a). No significant superiority of one technique over the other was found in a multivariable analysis. Importantly, the use of adjunctive IVUS during coronary angiography increased the chances of a successful RA-PCI by nearly 68% (Figure 1a). Considering other procedural indices, the radial access site, stent implantation, and fractional flow reserve (FFR) assessment during the angiogram were shown to be associated with technical success. Given the coronary lesion characteristics, bifurcation PCI was linked to higher odds for successful revascularization, whereas CTO (chronic total occlusion) lesions were found to be associated with a lower chance of achieving postprocedural TIMI 3 flow (Figure 1a). Furthermore, aspiration thrombectomy performed during the procedure as well as a higher usage of contrast dye and greater radiation exposure were associated with reduced odds for technical success (Figure 1a).

A more advanced age and the majority of concomitant comorbidities were associated with lower odds for postprocedural TIMI flow 3, except for hypertension being linked with 14% higher chances of successful revascularization (Figure 1b). Killip class IV (cardiogenic shock) or cardiac arrest at baseline were shown to be associated with decreased odds of postprocedural TIMI 3 flow, while direct transport, indicating the poor condition of a patient, was linked to higher odds of technical success (Figure 1b). Importantly, as shown in Figure 1c, the clinical presentation of coronary artery disease in patients undergoing RA-PCI had influence on the technical success rate (e.g., lower odds of achieving successful revascularization in NSTEMI/STEMI vs. SA patients).

Furthermore, all the periprocedural complications (MI, cardiac arrest, no-reflow phenomenon, coronary dissection, and stroke) and thrombolysis use were associated with lower chances of postprocedural TIMI 3 flow (Figure 1b).

## 4. Discussion

The present study supports the utilization of intravascular imaging to guide PCIs performed with concomitant rotational atherectomy. The notion is supported by the fact that postprocedural TIMI 3 flow was significantly more prevalent in IVUS- or OCT-guided RA-PCIs compared to angiography-guided procedures. Importantly, both the imaging modalities were independently associated with successful revascularization. Additionally, multivariable analysis revealed that among other factors linked to postprocedural TIMI 3 flow were younger age, bifurcation lesion, and prior revascularization attempts, whereas a worse clinical condition at baseline (Killip IV, presentation of acute coronary syndromes), greater procedural complexity (CTO lesion, more contrast and radiation used during PCI), and the incidence of periprocedural complications were associated with lower odds of the successful procedure.

Intravascular imaging modalities permit preprocedural lesion assessment, including an estimation of the true vessel size, based on which an optimal stent length and diameter can be chosen. With regard to RA-PCIs, this also allows for the selection of an optimal burr-to-artery ratio for appropriate calcified plaque modification without escalating the risk of periprocedural complications such as coronary artery dissection and perforation [25,26]. This might be particularly important not only for calcified de novo lesions, but also for RA-PCIs performed in stented segments [27]. The postprocedural assessment improves stent expansion and apposition as well as the detection of procedure-related complications. Although the utility of intravascular imaging depends on patients’ cohort characterization, the improvements of long-term clinical outcomes are generally well supported, particularly in severely calcified and complex lesions [14,16,28,29,30]. However, some studies encompassing both an all-comers cohort of patients and specific populations did not find significant improvements in the long-term endpoints (including MACE, target vessel revascularization, and definite stent thrombosis) following the introduction of intravascular imaging [31,32]. This is most likely driven by the lower complexity of PCIs (e.g., less severe calcifications), which reduces the potential benefits of intravascular imaging modalities. Since heavily calcified lesions are associated with stent underexpansion and the risk of in-stent restenosis, treatment of these particular lesions with the IVUS or OCT guidance yields most clinical benefits [33,34]. The armamentarium of calcium-modifying techniques is constantly expanding, and one can assume that the role of intravascular imaging modalities to guide these procedures will continue to increase in significance [35,36,37].

In the present analysis, the use of IVUS or OCT to guide RA-PCIs increased the odds for the postprocedural TIMI 3 flow by 67% and 66%, respectively. The technical success of the procedure defined as the achievement of TIMI 3 flow is a primary objective of all PCIs and has been shown to be an important predictor of improved clinical outcomes, including lower rates of in-hospital and long-term complications [38]. No difference between IVUS and OCT was found with regard to the achievement of successful revascularization. On the contrary, most of the published data comparing the efficacy of IVUS and OCT in the treatment of heavily calcified coronary arteries have shown the superiority of OCT over IVUS in terms of the periprocedural outcomes [16,17,38]. Kobayashi et al. reported greater stent expansion in OCT-guided RA-PCIs as compared to IVUS-guided RA-PCIs, concluding that RA with the guidance of OCT may be ideal for treating calcified coronary lesions [17]. Greater stent expansion following PCI guided with OCT was reported by other authors as well [16,39]. This is presumably driven by the higher resolution and more precise calcium visualization provided by OCT, as it detects calcium as signal-poor areas with well-delineated sharp borders [40]. On the contrary, IVUS does not permit the exact measurement of the calcium thickness given the fact that ultrasound signals are reflected by the calcium surface [41]. Therefore, OCT-guided RA-PCI enables more efficient calcium fracturing and facilitates optimal stent expansion. However, the immediate procedural success remains comparable, and the findings regarding long-term outcomes (the incidence of target lesion failure (TLR) and major adverse cardiovascular events (MACE)) remain inconsistent [16,17]. Therefore, the potential superiority of OCT in the treatment of severely calcified lesions warrants further studies.

Other findings of the present analysis are in line with the heretofore published data. In general, a younger age, prior revascularization attempts, and FFR are factors linked with better clinical outcomes, whereas a higher lesion complexity, worse patient clinical status at baseline, and procedural complications may have a detrimental influence on PCI outcomes [2,5,42,43].

## 5. Limitations

The present study has several limitations that need to be acknowledged when interpreting its results. First, this is a retrospective analysis with inherent limitations in the study design. It lacks randomization; therefore, one cannot exclude the influence of patient selection biases and unmeasured confounding factors on the final results. Second, the choice of intravascular imaging device was left entirely at the discretion of an operator, which could have also imposed a selection bias. Third, the number of patients undergoing OCT-guided RA-PCI was relatively small, which could have impacted the significance of the results. Fourth, due to the registry-based population cohort, certain data concerning RA-PCI were not available, including further follow-ups encompassing long-term outcomes of the RA-PCIs. Fifth, the data entered by operators were not double-checked for potential discrepancies with medical documentation. Lastly, in such a heterogenous population of patients (stable angina, unstable angina, NSTEMI, and STEMI), TIMI 3 flow might not be an equally adequate indicator of a successful procedure across the entire spectrum of patients, e.g., STEMI patients may have a large residual thrombus burden even following thrombectomy, which is a source of distal micro-embolization affecting postprocedural TIMI flow and long-term prognosis [44]. This is not observed in stable patients undergoing uncomplicated PCIs. The last issue is that the life span of the registry appears so long that technical changes could have modified the prevalence of using imaging modalities and even the clinical results.

## 6. Conclusions

In conclusion, the results of the present study indicate that intravascular imaging guidance (IVUS or OCT) during RA-PCIs is an independent predictor of a successful procedure (as defined by the achievement of postprocedural TIMI 3 flow); however, due to several limitations concerning the current analysis, the presented results should be considered as suggestive or supporting data. More studies are needed to evaluate the roles of intravascular imaging modalities in specific subsets of patients, including those treated with new emerging technologies.

## Figures and Tables

**Table 1:** Baseline characteristics of patients treated with percutaneous coronary intervention and rotablation.

	Total	Non-IVUS-OCT	IVUS-OCT	*p*-Value	Non-IVUS	IVUS	*p*-Value	Non-OCT	OCT	*p*-Value
	N = 6522	N = 5734	N = 788		N = 5814	N = 708		N = 6436	N = 86	
Age, years	72.1 ± 9.1 72 (66; 79)	72.1 ± 9.1 72 (66; 79)	71.4 ± 8.3 71 (66; 78)	0.03	72.1 ± 9.1 72 (66; 79)	71.5 ± 8.4 72 (66; 78)	0.07	72.1 ± 9.1 72 (66; 79)	71 ± 7.9 71 (66; 77)	0.29
Gender, males	2028 (31.2)	1812 (31.7)	216 (27.6)	0.01	1835 (31.7)	193 (27.4)	0.02	2002 (31.2)	26 (30.9)	0.96
Diabetes mellitus	4367 (67)	3874 (67.6)	493 (62.6)	0.005	3926 (67.5)	441 (62.3)	0.005	4311 (67)	56 (65.1)	0.71
Prior stroke	6265 (96.1)	5510 (96.1)	755 (95.8)	0.71	5587 (96.1)	678 (95.8)	0.67	6183 (96.1)	82 (95.4)	0.73
Prior MI	3416 (52.4)	3019 (52.7)	397 (50.4)	0.23	3066 (52.7)	350 (49.4)	0.10	3368 (52.3)	48 (55.8)	0.59
Prior PCI	2911 (44.6)	2556 (44.6)	355 (45.1)	0.81	2587 (44.5)	324 (45.8)	0.52	2878 (44.7)	33 (38.4)	0.28
Prior CABG	5701 (87.4)	5019 (87.5)	682 (86.6)	0.43	5093 (87.6)	608 (85.9)	0.19	5623 (87.4)	78 (90.7)	0.36
Smoking	5516 (84.6)	4842 (84.4)	674 (85.5)	0.43	4913 (84.5)	603 (85.2)	0.64	5440 (84.5)	76 (88.4)	0.33
Psoriasis	15 (0.2)	13 (0.2)	2 (0.3)	0.43	13 (0.2)	2 (0.3)	0.76	15 (0.2)	0 (0)	0.65
Arterial hypertension	1654 (25.4)	1422 (24.8)	232 (29.4)	0.006	1458 (25.1)	196 (27.7)	0.14	1618 (25.1)	36 (41.9)	0.001
Kidney disease	5760 (88.3)	5071 (88.4)	689 (87.4)	0.41	5142 (88.4)	618 (87.3)	0.37	5686 (88.4)	74 (86.1)	0.51
COPD	6289 (96.4)	5537 (96.6)	752 (95.4)	0.11	5613 (96.5)	676 (95.5)	0.15	6208 (96.5)	81 (94.2)	0.26

Data are presented as mean ± SD or median [Q1; Q3] and counts (percentages). IVUS, intravascular ultrasound; OCT, optical coherence tomography; MI, myocardial infarction; PCI, percutaneous coronary intervention; CABG, coronary artery bypass grafting; COPD, chronic obstructive pulmonary disease.

**Table 2:** Clinical characteristics of patients treated with percutaneous coronary intervention and rotablation.

	Total	Non-IVUS-OCT	IVUS-OCT	*p*-Value	Non-IVUS	IVUS	*p*-Value	Non-OCT	OCT	*p*-Value
	N = 6522	N = 5734	N = 788		N = 5814	N = 708		N = 6436	N = 86	
Clinical presentation										
- Acute heart failure	16 (0.3)	14 (0.2)	2 (0.3)	<0.001	15 (0.3)	1 (0.1)	<0.001	15 (0.2)	1 (1.2)	0.58
- Cardiac arrest	15 (0.2)	13 (0.2)	2 (0.3)		13 (0.2)	2 (0.3)		15 (0.2)	0 (0)	
- Chronic heart failure	114 (1.8)	87 (1.5)	27 (3.4)		89 (1.5)	25 (3.5)		112 (1.7)	2 (2.3)	
- Congenital heart defect	6 (0.1)	5 (0.1)	1 (0.1)		5 (0.1)	1 (0.1)		6 (0.1)	0 (0)	
- NSTEMI	829 (12.7)	743 (13)	86 (10.9)		749 (12.9)	80 (11.3)		823 (12.8)	6 (7)	
- Other	43 (0.7)	41 (0.7)	2 (0.3)		41 (0.7)	2 (0.3)		43 (0.7)	0 (0)	
- Stable angina	3672 (56.3)	3160 (55.1)	512 (65)		3209 (55.2)	463 (65.4)		3621 (56.3)	51 (59.3)	
- STEMI	532 (8.2)	501 (8.7)	31 (3.9)		506 (8.7)	26 (3.7)		524 (8.1)	8 (9.3)	
- Unstable angina	1295(19.9)	1170 (20.4)	125 (15.9)		1187 (20.4)	108 (15.3)		1277 (19.8)	18 (20.9)	
Killip class, mean	1.1 ± 0.4	1.1 ± 0.4	1.1 ± 0.3	0.17	1.1 ± 0.4	1.1 ± 0.3	0.35	1.1 ± 0.4	1 ± 0	0.21
	1 (1; 1)	1 (1; 1)	1 (1; 1)		1 (1; 1)	1 (1; 1)		1 (1; 1)	1 (1; 1)	
Killip class										
- I	1765 (92.9)	1609 (92.8)	147 (94.2)	0.31	1634 (92.9)	122 (93.1)	0.29	1730 (92.8)	26 (100)	0.57
- II	96 (5.1)	87 (5)	9 (5.8)		87 (5)	9 (6.9)		96 (5.2)	0 (0)	
- III	18 (1)	18 (1)	0 (0)		18 (1)	0 (0)		18 (1)	0 (0)	
- IV	20 (1.1)	20 (1.2)	0 (0)		20 (1.1)	0 (0)		20 (1.1)	0 (0)	
Killip class IV	20 (1.1)	20 (1.2)	0 (0)	0.4	20 (1.1)	0 (0)	0.39	20 (1.1)	0 (0)	1
Cardiac arrest at baseline	14 (0.6)	13 (0.6)	1 (0.5)	0.85	13 (0.6)	1 (0.5)	0.96	14 (0.6)	0 (0)	0.7
Hypothermia at baseline	2505 (99.9)	2290 (99.9)	215 (100)	0.66	2316 (99.9)	189 (100)	0.69	2478 (99.9)	27 (100)	0.88
Direct transport	2461 (98.2)	2249 (98.1)	212 (98.6)	0.62	2275 (98.1)	186 (98.4)	0.79	2434 (98.2)	27 (100)	0.48

Data are presented as median [Q1; Q3] and counts (percentages). IVUS, intravascular ultrasound; OCT, optical coherence tomography; NSTEMI, non-ST-elevation myocardial infarction; STEMI, ST-elevation myocardial infarction.

**Table 3:** Vascular access, coronary angiography, and periprocedural pharmacotherapy.

	Total	Non-IVUS-OCT	IVUS-OCT	*p*-Value	Non-IVUS	IVUS	*p*-Value	Non-OCT	OCT	*p*-Value
	N = 6522	N = 5734	N = 788		N = 5814	N = 708		N = 6436	N = 86	
Vascular access										
- Femoral	2149 (33)	1850 (32.3)	299 (38)	0.005	1879 (32.4)	270 (38.2)	0.006	2118 (33)	31 (36.5)	0.78
- Other	97 (1.5)	84 (1.5)	13 (1.7)		85 (1.5)	12 (1.7)		96 (1.5)	1 (1.2)	
- Radial	4265 (65.5)	3791 (66.2)	474 (60.3)		3840 (66.2)	425 (60.1)		4212 (65.6)	53 (62.4)	
Coronary angiography										
- Separate LMCA	422 (16.8)	344 (15)	78 (36.3)	<0.001	349 (15.1)	73 (38.6)	<0.001	417 (16.8)	5 (18.2)	0.98
- MVD	1254 (50)	1161 (50.7)	93 (43.3)		1175 (50.7)	79 (41.8)		1240 (50)	14 (51.9)	
- SVD	830 (33.1)	787 (34.3)	43 (20)		794 (34.3)	36 (19.1)		822 (33.2)	8 (29.6)	
ASA during PCI	4227 (64.8)	3734 (65.1)	493 (62.6)	0.16	3783 (65.1)	444 (62.7)	0.23	4174 (64.9)	53 (61.6)	0.57
UFH during PCI	965 (14.8)	861 (15)	104 (13.2)	0.2	869 (15)	96 (13.6)	0.34	956 (14.9)	9 (10.5)	0.29
LMWH during PCI	6382 (97.9)	5612 (97.9)	770 (97.7)	0.78	5692 (97.9)	690 (97.5)	0.44	6297 (97.8)	85 (98.8)	0.53
Bivalirudin during PCI	17 (0.26)	10 (0.17)	7 (0.89)	<0.001	11 (0.19)	6 (0.85)	0.001	15 (0.23)	2 (2.33)	<0.001
P2Y_12_ during PCI	3835 (58.8)	3395 (59.2)	440 (55.8)	0.66	3442 (59.2)	393 (55.5)	<0.001	3785 (58.8)	50 (58.1)	0.67

Data are presented as counts (percentages). IVUS, intravascular ultrasound; OCT, optical coherence tomography; LMCA, left main coronary artery; MVD, multi-vessel disease; SVD, single-vessel disease; PCI, percutaneous coronary intervention; CTO, chronic total occlusion; DEB, drug-eluting balloon; FFR, fractional flow reserve; ASA, acetyl-salicylic acid; UFH, unfractionated heparin; LMWH, low-molecular-weight heparin; TIMI, thrombolysis in myocardial infarction.

**Table 4:** Procedural indices.

	Total	Non-IVUS-OCT	IVUS-OCT	*p*-Value	Non-IVUS	IVUS	*p*-Value	Non-OCT	OCT	*p*-Value
	N = 6522	N = 5734	N = 788		N = 5814	N = 708		N = 6436	N = 86	
Preprocedural TIMI 0/1 flow	1195 (18.9)	1084 (19.6)	111 (14.3)	<0.001	1088 (19.4)	107 (15.3)	0.009	1187 (19.1)	8 (9.5)	0.03
Postprocedural TIMI 3 flow	6.136 (96.8)	5365 (96.6)	771 (98.7)	<0.0001	5441 (96.6)	695 (98.9)	<0.001	6054 (96.8)	82 (97.6)	0.68
PCI within CTO	346 (5.3)	305 (5.3)	41 (5.2)	1.0	309 (5.3)	37 (5.2)	0.92	340 (50.3)	6 (7)	0.49
PCI within bifurcation	973 (14.9)	732 (12.8)	241 (30.6)	<0.001	743 (12.8)	230 (32.5)	<0.001	960 (14.9)	13 (15.1)	0.96
PCI with stent	5927 (90.8)	5208 (90.8)	719 (91.2)	0.70	5278 (90.8)	649 (91.7)	0.44	5851 (90.9)	76 (88.4)	0.42
PCI with DEB	57 (0.9)	47 (0.9)	10 (1.4)	0.18	50 (0.9)	7 (1.1)	0.69	53 (0.9)	4 (4.9)	<0.001
PCI with DES	5900 (90.5)	5183 (90.4)	717 (91)	0.59	5251 (90.3)	649 (91.7)	0.25	5826 (90.5)	74 (86.1)	0.16
PCI with BRS	14 (0.2)	10 (0.2)	4 (0.5)	0.06	13 (0.2)	1 (0.1)	0.65	10 (0.2)	4 (4.7)	<0.001
PCI with BMS	23 (0.4)	22 (0.4)	1 (0.1)	0.25	22 (0.4)	1 (0.1)	0.31	22 (0.3)	1 (1.2)	0.2
FFR during PCI	6461 (99.1)	5684 (99.1)	777 (98.6)	0.15	5764 (99.1)	697 (98.5)	0.07	6375 (99.1)	86 (100)	0.36
Aspiration thrombectomy during PCI	37 (0.57)	33 (0.58)	4 (0.51)	0.81	33 (0.57)	4 (0.56)	0.99	37 (0.57)	0 (0)	0.48
Contrast amount, mL	205 ± 89 2.0 (1.5; 2.5)	202 ± 0.87 2.0 (1.5; 2.5)	2.31 ± 0.94 2.0 (1.7; 2.8)	<0.001	2.02 ± 0.87 2.0 (1,5; 2.5)	2.31 ± 0.95 2.1 (1.7; 2.8)	<0.001	2.05 ± 0.89 2.0 (1.5; 2.5)	2.30 ± 0.96 2.0 (1.8; 2.7)	0.01
Radiation exposure, Gy	1.47 ± 1.17 1.2 (0.7; 1.9)	1.45 ± 1.16 1.1 (0.7; 1,9)	1.59 ± 1.22 1.3 (0.8; 2.1)	0.003	1.46 ± 1.16 1.1 (0.7; 1.9)	1.59 ± 1.22 1.3 (0.8; 2.1)	0.005	1.47 ± 1.16 1.2 (0.7; 1.9)	1.66 ± 1.38 1.4 (0.8; 2.0)	0.21

Data are presented as mean ± SD or median [Q1; Q3] and counts (percentages). BMS, bare-metal stent; BRS, bioresorbable stent; CTO, chronic total occlusion; DEB, drug-eluting balloon; DES, drug-eluting stent; FFR, fractional flow reserve; IVUS, intravascular ultrasound; TIMI, thrombolysis in myocardial infarction; OCT, optical coherence tomography; PCI, percutaneous coronary intervention.

**Table 5:** Percutaneous coronary intervention-related periprocedural complications.

	Total	Non-IVUS-OCT	IVUS-OCT	*p*-Value	Non-IVUS	IVUS	*p*-Value	Non-OCT	OCT	*p*-Value
	N = 6522	N = 5734	N = 788		N = 5814	N = 708		N = 6436	N = 86	
Death during procedure	6500 (99.7)	5713 (99.6)	787 (99.9)	0.28	5793 (99.6)	707 (99.9)	0.34	6414 (99.7)	86 (100)	0.59
MI	6492 (99.5)	5706 (99.5)	786 (99.8)	0.36	5786 (99.5)	706 (99.7)	0.46	6407 (99.6)	85 (98.8	0.33
No reflow	6472 (99.2)	5688 (99.2)	784 (99.5)	0.37	5767 (99.2)	705 (99.6)	0.27	6387 (99.2)	85 (98.84)	0.67
Bleeding at the puncture site	6512 (99.9)	5726 (99.9)	786 (99.8)	0.44	5806 (99.9)	706 (99.7)	0.35	6426 (99.8)	86 (100)	0.71
Cardiac arrest	6491 (99.5)	5705 (99.5)	786 (99.8)	0.34	5785 (99.5)	706 (99.7)	0.43	6405 (99.5)	86 (100)	0.59
Allergic reaction	6519 (100)	5731 (100)	788 (100)	0.52	5811 (100)	708 (100)	0.55	6433 (200)	86 (100)	0.84
CAP	6456 (99)	5680 (99.1)	776 (98.5)	0.13	5760 (99.1)	696 (98.3)	0.05	6370 (99)	86 (100)	0.35

Data are presented as counts (percentages). CAP, coronary artery perforation; IVUS, intravascular ultrasound; OCT, optical coherence tomography; MI, myocardial infarction.

## Data Availability

Upon special request.
